# Plant–plant communication in variety mixtures plays on disease susceptibility and immunity

**DOI:** 10.1093/jxb/erab377

**Published:** 2021-09-30

**Authors:** Marie-Odile Bancal

**Affiliations:** 1 AgroParisTech, University Paris-Saclay, France; 2 INRAE, ECOSYS, UMR 1402, F-78350 Thiverval-Grignon, France

**Keywords:** Disease, immunity, intraspecific mixture, neighbour, plant, plant interactions, rice, wheat

## Abstract

This article comments on:

**Pélissier R, Buendia L, Brousse A, Temple C, Ballini E, Fort F, Violle C, Morel JB.** 2021. Plant neighbour-modulated susceptibility to pathogens in intraspecific mixtures. Journal of Experimental Botany **72**, 6570–6580.

**Intraspecific mixtures are a well-known means to modulate epidemics in crops, but knowledge of the immunity they induce is scarce. Pélissier *et al.* (2021a) selected pairs of susceptible wheat or rice cultivars cross-modulating disease severity and showed that belowground interactions were involved in communicating infection. Healthy neighbours could initiate significant modulations of transcription of basal immunity genes after pathogen inoculation. No general rule was observed between pathosystems, but the demonstration of the effect of healthy neighbours on disease susceptibility and immunity in adjacent plants is a key finding as we strive to understand health in varietal mixtures.**

## Biodiversity: an insurance for plant health?

In crops, maintaining plant health is crucial, as climate change and decreased availability of pesticides may enhance opportunities for pests and diseases (Barot *et al.*, 2017). A large consensus states that higher natural or cultivated biodiversity increases productivity because functional complementarity and redundancy enhance resilience to environmental fluctuations. In most but not all cases, complex but beneficial biological regulations between trophic or scale levels emerge from ecosystem biodiversity (Tilman *et al.*, 2014). Mixing varieties is an easy-to-use and flexible means to adapt annual crops to identified patterns of multistress (Grettenberger and Tooker, 2015). Identifying and quantifying the main mechanisms involved as biodiversity increases is a major prerequisite for managing assemblies in agroecology.

Biodiversity attenuates the population response to biotic and abiotic stresses by mobilizing common mechanisms (Tilman *et al.*, 2014; Barot *et al.*, 2017; Rolfe *et al.*, 2019): escape by phenology or architecture; protection by genetic barriers (‘dilution’ of susceptibility among resistant plants); niche complementarity for resources; microclimate modification; and finally plant immunity. All these mechanisms interplay directly or indirectly to achieve a better crop fitness. The result depends on whether competitive or facilitative mechanisms dominate and on the environmental conditions (Tilman *et al.*, 2014; Rolfe *et al.*, 2019). While these mechanisms support similar ranges of yield gains, crop management considerations generally favour genetic complementarity and/or remote enhancement of immunity responses in variety mixtures (Grettenberger and Tooker, 2015; Gibson and Nguyen, 2020), even though asymmetric access to resources may occur (Pélissier *et al.*, 2021b).

Variety mixtures rely mainly on complementarity between genotypes which are susceptible and resistant to the more frequent and damaging pests/diseases (Grettenberger and Tooker, 2015; Barot *et al.*, 2017). Alternatively, remote plant immunity involves manipulating a sequence of events from the recognition of a danger/stranger, its transduction, and the associated induced physiological responses (Rolfe *et al.*, 2019; Pélissier *et al.*, 2021b).

Near-neighbour crop communications, as premunition of exposure to pathogens and/or priming by emission of a defence-related compound, were identified long ago. From the 2000s, the key role of immunity in population health largely extended to plant–plant interactions during infection. Plants interact either directly through chemical exchanges, namely exudates and volatile organic compounds (VOCs), or indirectly through the microbiome, a network of above- or belowground microorganisms so closely associated with plants that it behaves like a meta-organism, a holobiome (Trivedi *et al.*, 2020; Sharifi and Ryu, 2021).

Pests or pathogens largely induce host plant immunity by well-known phytohormones including salicylic acid, ethylene, and jasmonic acid (Benevenuto *et al.*, 2020; Trivedi *et al.*, 2020; Sharifi and Ryu, 2021), all of which may prime neighbours (Ninkovic *et al.*, 2021). Interestingly, kin and stranger recognition also crosstalk with defence pathways (Liu *et al.*, 2020; Sharifi and Ryu, 2021), using root exudates (Anten and Chen, 2021) or leaf volatiles (Karban *et al.*, 2013), diverting carbon towards beneficial anticipation responses (Anten and Chen, 2021). Interplant cues and signals have been found to be central in herbivory (Pélissier *et al.*, 2021b). However, the role of plant immunity in regulating disease development in variety mixtures has certainly been understudied. Furthermore, if priming is a well-established mechanism to anticipate stresses, plant–plant communication in healthy conditions was rarely advocated, especially in conspecific plant populations (Ninkovic *et al*., 2021; Pélissier *et al.*, 2021b).

## Do healthy conspecific neighbours enhance plant resistance and immunity?

In their study published in this issue, Pélissier *et al.* (2021a) investigated whether mixing pairs of susceptible varieties of wheat or rice may limit severity and increase immunity, even if the focal receiver plant is neighboured by healthy plants. To unravel plant–plant interactions and focus on immunity only, they set up a glasshouse experiment with both susceptible focal and neighbour plants. The experimental design, moreover, limited plant competition for resources and pathogen dispersal. The originality of the study is the isolation of basal plant immunity from other defence mechanisms to pinpoint the importance of immunity in the health of conspecific plant populations.

Pélissier *et al.* (2021a) found that it is possible to select pairs of varieties that significantly cross-protected against rust in wheat or blast in rice, compared with monocrops. Given that the associated varieties were equally susceptible, the mixture advantage does not rely directly on resistance genes to the inoculated pathogens. It confirms previous studies showing that biodiversity mainly ameliorates plant health (Gibson and Nguyen, 2020). However, Pélissier *et al.* did not generalize their observation. Inoculation of mixtures with other pathogens, even those with a closely related lifestyle, showed either no change or an increased severity in both focal and neighbour plants. The literature previously pointed out the lack of genericity of pathogen responses to biodiversity (Rolfe *et al.*, 2019; Liu *et al.*, 2020), rendering its use in field crops submitted to multiple and unpredictable stresses difficult (Berens *et al.*, 2019; Landi, 2020).

Using the same wheat or rice variety pairs, Pélissier *et al.* (2021a) tried to elucidate by which mechanisms conspecific plants interplay. In both cases, they showed that varieties communicated directly by a belowground chemical interaction between kin roots. Root physical contact was needed in wheat mixtures, but not in rice. Surprisingly, neither the soil microbiome nor the inoculation of neighbours was necessary for this interplay. The originality of the study was to state that healthy and inoculated neighbour plants modified focal plant susceptibility to the same extent, suggesting that specific kin recognition between cultivars was enough to enhance plant health (Gibson and Nguyen, 2020). Indeed, the key role of plant exudates in plant–plant communication had already been shown (Rolfe *et al.*, 2019; Liu *et al.*, 2020), and the reshaping of the microbiome after defence priming was also recently demonstrated (Trivedi *et al*., 2020). This study moreover suggests that early plant–plant interactions in varietal mixtures may reshape plant health status only due to the proximity of close but distinct genotypes and without priming. This mechanism has not yet been exemplified, maybe because it was hidden by longer term interactions (Trivedi *et al*., 2020), or because other plant–plant interactions interfere, such as resource competition or multistress responses (Benevenuto *et al.*, 2020; Landi, 2020).

Finally, basal immunity was a good candidate to trigger these processes. In healthy rice plants, the basal immunity was enhanced by mixture; this effect was even strengthened by inoculation, suggesting that the changes in susceptibility relate to those in the basal immunity. Conversely, in wheat, the basal immunity of healthy plants was unchanged by mixture, while inoculation modulated the immunity of each variety differently. Consequently, changes in basal immunity could not explain changes in wheat mixture susceptibility. In varietal mixtures, the link between decreased susceptibility and increased basal immunity may not be a general rule.

## Perspectives for crop health management

This is the first study pointing out that mixing close relatives enhances crop health, even without priming, by using root chemical signalling. Interestingly, this imaginative experimental design revealed that an underlying communication between healthy kin plants, even if weak, may enhance the population’s health, by modulating a complex network of interactions (Liu *et al.*, 2020). This is a key finding to avoid confusion of effects when setting up experimental designs involving inter- and intraspecific mixtures under multistresses (Landi, 2020). Furthermore, it opens up the way to search for new chemical traits, based on early plant–plant recognition, for assembly of varieties.

This study nevertheless raises some questions. (i) If belowground signals or cues were clearly involved, aerial VOCs may also interplay (Ninkovic *et al.*, 2021), which the experimental design did not show, preventing pathogen but not VOC movements. (ii) Short-term responses to mixtures may be relayed by longer term soil microbiome changes (Rolfe *et al.*, 2019; Benevenuto *et al.*, 2020). (iii) The variety relatedness, which is usually high (Grettenberger and Tooker, 2015), was not examined, although it is key information for the future assembly of varieties, even susceptible ones (Gibson and Nguyen, 2020). This last point particularly challenges the link with agricultural practices that largely use complementary resistance profiles in mixtures (Barot *et al.*, 2017). More generally, (iv) these effects must be confirmed under natural conditions, with varietal mixtures subjected to multiple stresses: climate (Berens *et al.*, 2019; Benevenuto *et al.*, 2020), soil and its biotic history (Sharifi and Ryu, 2021), nutrition (Landi, 2020; Liu *et al.*, 2020) and/or pests (Liu *et al.*, 2020), which all interact with immunity towards a given outcome.

This study provides a renewed insight into interactions between closely related plants, putting forward an underlying mechanism of ecosystem resilience that future research has to investigate in more depth for further applications in agroecology.
